# Death by Neurologic Criteria in Neonates Undergoing Extracorporeal Membrane Oxygenation: Extracorporeal Life Support Organization Registry Study, 2010–2023

**DOI:** 10.1097/PCC.0000000000003891

**Published:** 2026-01-12

**Authors:** Angelo Polito, Arthur Gavotto, Anne-Marie Guerguerian, Melania M. Bembea, Roberto Lorusso, Aparna Hoskote, Nicolas Joram, Akram M. Zaaqoq, Sung-Min Cho, Matteo Di Nardo, Lakshmi Raman, Ravi R. Thiagarajan, on behalf of the European Branch of the Extracorporeal Life Support Organization (Euro-ELSO) Working Group on Neurologic Monitoring and Outcome, Mirjana Cvetkovic

**Affiliations:** 1 Pediatric Intensive Care Unit, Department of Woman, Child, and Adolescent Medicine, Geneva University Hospital, Geneva, Switzerland.; 2 Faculty of Medicine, University of Geneva, Switzerland.; 3 PhyMedExp, CNRS, INSERM, U1046: Paediatric Intensive Care Unit, Woman’s, Mother’s and Children’s Department, Arnaud de Villeneuve Hospital, Montpellier University Hospital, Montpellier, France.; 4 Department of Critical Care Medicine, Research Institute, The Hospital for Sick Children, University of Toronto, Toronto, ON, Canada.; 5 Department of Anesthesiology and Critical Care Medicine, Johns Hopkins University School of Medicine, Baltimore, MD.; 6 Cardio-Thoracic Surgery Department, Maastricht University Medical Centre (MUMC), Maastricht, The Netherlands.; 7 Heart and Lung Division, Great Ormond Street Hospital for Children NHS Foundation Trust, London, United Kingdom.; 8 Pediatric Intensive Care Unit, Women’s, Children’s and Adolescent’s Department, Nantes University Hospital, Nantes, France.; 9 Department of Anesthesiology, Division of Critical Care, University of Virginia, Charlottesville, VA.; 10 Division of Cardiac Surgery, Department of Surgery, Johns Hopkins Hospital, Baltimore, MD.; 11 Division of Neurosciences Critical Care, Department of Neurology, Neurosurgery, Anesthesiology and Critical Care Medicine, Johns Hopkins Hospital, Baltimore, MD.; 12 Pediatric Intensive Care Department, Bambino Gesù Children’s Hospital, IRCCS, Rome, Italy.; 13 Department of Pediatrics, Section Critical Care Medicine, Children’s Medical Center at Dallas, The University of Texas Southwestern Medical Center at Dallas, Dallas, TX.; 14 Division of Cardiovascular Critical Care, Department of Cardiology, Boston Children’s Hospital, Boston, MA.

**Keywords:** death by neurologic criteria, hyperoxia, hypocapnia, neonatal extracorporeal membrane oxygenation

## Abstract

**Objectives:**

To identify factors associated with the development of death by neurologic criteria (DNC) in neonates treated with extracorporeal membrane oxygenation (ECMO).

**Design:**

Retrospective registry study.

**Setting:**

Data reported to the Extracorporeal Life Support Organization registry from 2010 to 2023.

**Patients:**

Neonates (≤ 28 d old) who were supported with ECMO, excluding those born before 37 weeks’ gestation or with missing gestational age data. The final cohort comprised 14,970 neonates.

**Interventions:**

None.

**Measurements and Main Results:**

DNC occurred in 70 neonates in the cohort (0.5%), accounting for 2% of overall mortality rate. Pre-ECMO factors associated with greater relative risk ratio (RRR) of DNC included pre-ECMO cardiac arrest (RRR, 2.64), pH less than 7.08 (25th percentile: RRR, 2.06), and cardiac support type (RRR, 2.04). On-ECMO, factors independently associated with DNC included pH less than 7.35 (25th percentile: RRR, 2.76), Pao_2_ greater than 162 mm Hg (75th percentile: RRR, 2.75), and central cannulation (RRR, 2.36). We failed to identify an association between relative change in Paco_2_ greater than 50% and DNC, but it correlated with other causes of death. Most DNC diagnoses (84%) occurred after 24 hours of ECMO.

**Conclusions:**

DNC is rarely diagnosed in neonatal ECMO cases. Both pre-ECMO and on-ECMO factors associated with DNC included pre-ECMO cardiac arrest, severe metabolic acidosis, and cannulation type. These findings underscore the importance of optimizing pre-ECMO and on-ECMO management and may indicate certain modifiable risk factors such as optimization of cardiopulmonary resuscitation and hyperoxia. Future research should explore preventive strategies and interventions to mitigate the risk of DNC in neonates receiving ECMO.

**RESEARCH IN CONTEXT**

- Extracorporeal membrane oxygenation (ECMO) provides a life-saving support for neonates with severe cardiorespiratory failure.
- ECMO is associated with substantial risks, particularly neurologic complications. Among these, brain death, or death by neurologic criteria (DNC).
- Identifying factors associated with the diagnosis of DNC is crucial to improve early recognition, refine surveillance strategies, and ultimately enhance care quality and effective communication with families.

**WHAT THIS STUDY MEANS**

- In the 2010–2023 Extracorporeal Life Support Organization dataset, DNC is rare in neonates supported with ECMO but may be under-diagnosed.
- Pre-ECMO factors associated with greater relative risk ratio of DNC include pre-ECMO cardiac arrest, severe metabolic acidosis, and cardiac support type; on-ECMO associated factors include pH less than 7.35, Pao_2_ greater than 162 mm Hg, and central cannulation.
- These findings may help generate hypotheses regarding clinical factors that warrant closer monitoring of pre-ECMO and on-ECMO management and highlight potential modifiable factors associated with DNC.

Extracorporeal membrane oxygenation (ECMO) provides life-saving support for neonates with severe cardiorespiratory failure (1). However, ECMO is associated with substantial risks, particularly neurologic complications (2). Among these complications, brain death, also referred to as death by neurologic criteria (DNC), is irreversible and can be diagnosed while the patient is still supported on ECMO, with significant clinical and ethical implications (3). The neonatal brain is particularly vulnerable to injury during episodes of critical illness due to its developmental immaturity. Contributing factors to DNC may include hemodynamic instability, coagulopathy, hypoxia-ischemia, and systemic inflammation and the physiologic disruptions caused by an extracorporeal circulation (3–7). To date, a number of Extracorporeal Life Support Organization (ELSO) studies of neonates supported with ECMO have explored factors associated mortality (3–5, 8), and the development of DNC in neonates remain insufficiently understood.

In this context, we consider that identifying the factors associated with the diagnosis of DNC in neonates supported with ECMO is crucial to improve early recognition, refine surveillance strategies and, ultimately, to enhance better care and effective communication with families (9, 10). Therefore, in this study, we first aimed to identify the frequency of DNC in neonates supported with ECMO, as recorded in the ELSO registry. Secondarily, we sought to identify potential factors associated with DNC in the dataset.

## METHODS

In this retrospective cohort study, we analyzed data obtained from the ELSO registry, 2010–2023. The ELSO registry collects information about patients and devices during support with ECMO and, as an agreement between member centers and ELSO, the organization allows the release of de-identified datasets for research purposes with organizational governance and approval. Therefore, no Institutional Review Board approval was necessary, but with all research handling patient data we followed out institutional practices and worked in accordance with the 1975 Declaration of Helsinki.

We used ELSO registry data for cases entered from January 1, 2010, to December 31, 2023. All newborns (≤ 28 d old) undergoing ECMO were included. We excluded neonates born before 37 weeks’ of gestational age (GA), as the clinical determination of DNC in the United States may be either invalid or unaccepted at this age (11). For patients with more than one ECMO run, only the last run was included.

### Study Variables

The ELSO registry collects pre-ECMO variables including patient age, comorbidities as defined by *International Classification of Diseases*, 9th Edition (ICD)-9 and *International Classification of Diseases*, 10th Edition (ICD-10), Clinical Modification (12) (**Table S1**, https://links.lww.com/PCC/C688), hemodynamic, and arterial blood gas (ABG) values. Pre-ECMO data are collected within the 6 hours preceding ECMO. Pre-ECMO Pao_2_ may be low in patients with congenital heart diseases (CHDs) regardless of the presence of respiratory insufficiency and was excluded from the analysis. Pre-ECMO support included mechanical circulatory support devices, respiratory support, use of vasoactive infusions, and IV medications. On-ECMO variables collected include cannulation sites, laboratory information collected close to 24 hours after ECMO initiation, complications, ECMO duration, and reason for ECMO discontinuation (i.e., DNC, other criteria for death or withdrawal, recovery, or transplant).

### Data Categorization and Outcome

The ICD-9 and ICD-10, Clinical Modification were used to create the “cardiac” diagnostic category including “myocarditis and cardiomyopathies,” “CHD,” and “other cardiac.” Other diagnostic categories included: meconium aspiration syndrome, congenital diaphragmatic hernia, asphyxia of the newborn, persistent pulmonary hypertension of the newborn, and sepsis/septic shock and “other,” regrouping patients that could not be assigned to one of these groups. Patients were classified in the “cervical cannulation” group if either the carotid artery or the jugular vein was one of the cannulation sites reported. Reason for ECMO discontinuation was categorized as “death by other causes,” meaning other criteria for death, if ECMO was discontinued because of complications, resource limitation, or poor prognosis (excluding DNC); as “transplant” if heart transplant, lung transplant, or ventricular assist device placement were performed. DNC is categorized as a neurologic complication (code 301) if it occurs before ECMO discontinuation.

### Statistical Analysis

The study was performed and analyzed following the Strengthening the Reporting of Observational Studies in Epidemiology statement (13). We were cognizant of prior research using the ELSO dataset and the overlap with previously reported factors associated with death (3–5, 8), and the journals requirements for such studies (14). Descriptive statistics were used to describe unadjusted patient characteristics, including count, proportions and percentages (%), and median (interquartile range [IQR]).

The exact timing of determining DNC is not available in the ELSO registry. Furthermore, as time from ECMO to DNC is arbitrary and depends on clinical and nonclinical factors, multinomial regression analyses looking at DNC, other (not DNC) deaths, transplant, and survivors (reference group) as primary outcome were used. Two separate multivariable models were used to analyze pre-ECMO and on-ECMO associations with DNC. Only patients with at least 24 hours of support were included in the on-ECMO model. The multivariable models were adjusted for a priori defined clinically relevant variables. Pre-ECMO data are collected within 6 hours preceding ECMO. For the pre-ECMO model, the following variables were included: weight (kg), pre-ECMO cardiac arrest, ECMO indication, ECMO mode, Apgar score at 5 minutes after birth, pH and Paco_2_, and previous ECMO run. On-ECMO variables are collected close to 24 hours after ECMO onset. For the on-ECMO model, the following variables were included: cannulation site (cervical vs. central), pH and Pao_2_, ECMO flow and the relative change (%) in Paco_2_ between the pre-ECMO period and after ECMO (i.e., calculated as the difference between the on-ECMO and pre-ECMO Paco_2_) divided by pre-ECMO Paco_2_, and all multiplied by 100, and categorized as greater than 50% (15). As variations in resources, expertise, and institutional protocols can influence patient outcomes, center volume over the whole period (low < 20, medium 20–49, and high ≥ 50) (16) was also added into the on-ECMO model. Only patients with at least 24 hours of support were included in the on-ECMO model. All continuous variables were tested for log-linearity assumption. In case of a nonlinear relationship, variables were divided into clinically relevant quartiles as appropriate. Reference values were determined according to the closest physiologic ranges. Variables with more than 20% missing data were excluded from the analysis. Post hoc sensitivity analysis was carried out to stress the robustness of the model: a multiple linear regression (MLR) model excluding transplant patients and an MLR model merging the “other death” and “transplant” categories of the dependent variable. Statistical significance was set at the two-tailed 0.05 level for all analyses. All analyses were performed using R-4.2.2 (R Foundation for Statistical Computing, Vienna, Austria) and Stata18 (StataCorp, College Station, TX) software (mlogit command for MLR models).

## RESULTS

During 2010–2023, there was a total of 19,833 neonates who received ECMO in the ELSO registry. Of these, 2744 had a GA less than 37 weeks, and 2119 had a missing GA and were excluded from the analysis. Consequently, the final study population consisted of 14,970 patients. A total of 279 unique centers reported at least one neonatal case to the ELSO registry during the study period, with the annual distribution of reporting centers detailed in **Table S2** (https://links.lww.com/PCC/C688).

Two thousand six hundred twenty-five neonates (18%) were reported to have had acute brain injury (ABI), the most common, not mutually exclusive, types of ABI included: seizures in 1158 (8%), intracranial hemorrhage (ICH) in 1367 (9%), ischemic stroke in 89 (1%), and DNC in 70 (0.5%). Among patients with DNC, ABI included seizures in 9 (13%), ICH in 16 (23%), and ischemic stroke in 27 (38%).

Six hundred forty-three patients (4%) underwent more than one ECMO run. Pre- and on-ECMO patients’ characteristics, ABG values, comorbidities, and support are shown in **Table 1** and **Table S3** (https://links.lww.com/PCC/C688). The median time spent on ECMO was 5.7 (IQR, 3.5–9.8). The majority of patients (10,644, 71%) were cannulated via cervical vessels, while 4,231 (29%) were centrally cannulated. Among neonates with cervical cannulation, 8444 (79.5%) were supported with venoarterial ECMO, and 2173 (20.5%) were supported with venovenous ECMO. For centrally cannulated neonates, 4027 (93.6%) underwent venoarterial ECMO, compared with 277 (6.4%) who were supported with venovenous ECMO.

**TABLE 1.** Characteristics of the Whole Study Population and According to Primary Outcome

Variables	Overall (*n* = 14,970)	Survived (*n* = 11,367)	Death by Neurologic Criteria (*n* = 70)	Other Deaths (*n* = 3,482)	Transplant (*n* = 51)
Sex (female %)	6,430 (43)	4,773 (42)	34 (49)	1,603 (46)	20 (39)
Age (d)	5.1 ± 6.2	4.7 ± 6.0	6.9 ± 7.0	6.2 ± 6.8	10.1 ± 7.3
Birth weight (kg)	3.2 (2.9–3.6)	3.3 (3.0–3.7)	3.3 (2.9–3.7)	3.1 (2.8–3.5)	3.2 (2.9–3.6)
Gestational age (wk)	39 (38–40)	39 (38–40)	39 (38–40)	39 (38–39)	39 (38–39)
Apgar 5 min > 6 (%)	9,056 (66)	6,775 (64)	48 (76)	2,190 (70)	43 (93)
Center volume *n* (%)					
< 20	12,224 (82)	9,198 (81)	63 (90)	2,917 (84)	46 (90)
20–49	2,746 (18)	2,169 (19)	7 (10)	565 (16)	5 (10)
> 50	0 (0)	0 (0)	3 (4)	87 (2)	0 (0)
Previous ECMO run	532 (4)	315 (3)	3 (4)	210 (6)	4 (8)
Diagnostic categories (%)^a^					
Cardiac disease	3,195 (21)	2,206 (19)	19 (27)	933 (27)	37 (76)
Meconium aspiration syndrome	2,375 (16)	2,235 (20)	6 (9)	134 (4)	0 (0)
Congenital diaphragmatic hernia	2,533 (17)	1,851 (16)	4 (6)	678 (20)	0 (0)
Asphyxia of the newborn	14 (0)	11 (0)	0 (0)	3 (0)	0 (0)
Persistent pulmonary hypertension	1,435 (10)	1,231 (11)	5 (7)	199 (6)	0 (0)
Sepsis/septic shock	342 (2)	204 (2)	9 (13)	129 (4)	0 (0)
Pre-ECMO cardiac arrest	2,738 (18)	1,814 (16)	27 (39)	880 (26)	17 (34)
ECMO indications (%)					
Cardiac	4,634 (31)	3,189 (28)	32 (46)	1,373 (39)	40 (78)
Extracorporeal cardiopulmonary resuscitation	1,427 (10)	908 (8)	13 (19)	496 (14)	10 (20)
Pulmonary	8,909 (60)	7,270 (64)	25 (36)	1,613 (46)	1 (2)
First ECMO mode (%)					
Venoarterial	12,471 (84)	9,244 (82)	66 (96)	3,110 (90)	51 (100)
Venovenous	2,450 (16)	2,086 (18)	3 (4)	361 (10)	0 (0)
Cervical cannulation	10,644 (71)	8,380 (74)	42 (60)	2,198 (63)	24 (47)
ECMO duration (d)	8.0 ± 8.2	7.3 ± 7.0	3.7 ± 3.1	10.3 ± 10.9	12.9 ± 13.7

ECMO = extracorporeal membrane oxygenation.

a Not mutually exclusive.

Values are expressed as *n* (%), medians (interquartile range), or means ± sd.

**Figure 1.**
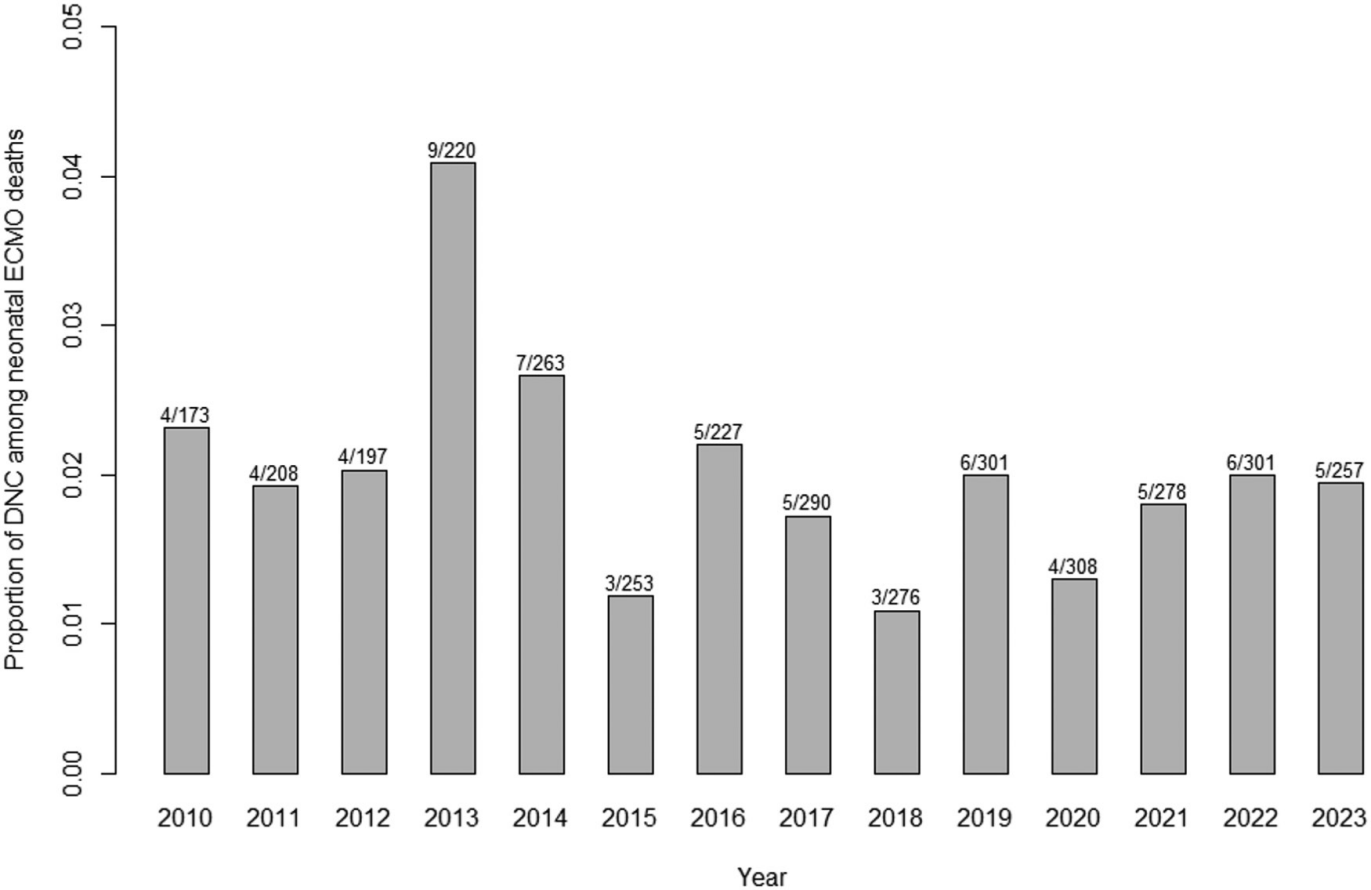
Proportion of death by neurologic criteria (DNC) among deceased patients over the period 2010–2023. ECMO = extracorporeal membrane oxygenation.

The reason for ECMO discontinuation was DNC in 70 patients (0.5%), other criteria for death in 3,482 patients (23%), recovery in 11,367 (76%), and transplantation in 51 patients (< 1%). Diagnosis of neonatal DNC, vs. not, was associated with lower pH, lower Paco_2_, lower bicarbonate, and higher lactate levels before ECMO cannulation. DNC was also associated with lower on-ECMO pH; however, we failed to identify an association with on-ECMO Paco_2_ value. Furthermore, a higher percentage of patients with DNC, compared with those not, had pre-ECMO cardiac arrest. Most patients progressing to determination of DNC (59/70, 84%) were diagnosed after 24 hours of ECMO. The occurrence of DNC rate over 2010–2023 shown in **Figure 1**.

Results of regression analyses are shown in **Tables 2** and **3**. In the pre-ECMO model, pre-ECMO cardiac arrest (relative risk ratio [RRR], 2.59), pH less than 7.08 before ECMO (25th percentile: RRR, 2.34), and cardiac support type (RRR, 1.95) were each independently associated with subsequent DNC. Among patients with more than 24 hours of ECMO support, pH less than 7.35 (25th percentile: RRR, 2.76), Pao_2_ greater than 162 mm Hg (75th percentile: RRR, 2.75), and central cannulation (RRR, 2.36) were each independently associated with subsequent DNC. We failed to identify an association between a relative change in Paco_2_ greater than 50% (occurring between the pre-ECMO period and 24 hr after ECMO initiation) and subsequent DNC. However, such a change in Paco_2_ was associated with other non-DNC deaths (RRR, 1.44).

**TABLE 2.** Pre-Extracorporeal Membrane Oxygenation Model of Multinomial Logistic Regression Analysis Comparing Death by Neurologic Criteria, Other Non-Death by Neurologic Criteria Deaths, and Transplant With Survivors As the Reference Group

Variables	DNC (*n* = 70)		Other Non-DNC Death (*n* = 2724)		Transplant (*n* = 34)	
	RRR	*p*	RRR	*p*	RRR	*p*
Pre-ECMO cardiac arrest	2.59 (1.37–4.90)	**0.003**	1.29 (1.13–1.47)	**< 0.001**	1.44 (0.58–3.53)	0.430
Support type						
Pulmonary	Reference		Reference			
Cardiac	1.95 (1.03–3.68)	**0.040**	1.74 (1.56–1.94)	**< 0.001**	—	—
Extracorporeal cardiopulmonary resuscitation	1.07 (0.40–2.82)	0.895	1.65 (1.37–1.99)	**< 0.001**	—	—
ECMO mode						
Venoarterial	Reference		Reference			
Venovenous	0.36 (0.11–1.22)	0.101	0.70 (0.60–0.80)	**< 0.001**	—	—
Apgar 5 min						
7–10^a^	Reference		Reference		Reference	
0–6	0.84 (0.45–1.58)	0.588	0.98 (0.89–1.09)	0.792	0.20 (0.03–1.44)	0.109
Weight (kg)						
≥ 2.9	Reference		Reference		Reference	
< 2.9^b^	1.24 (0.69–2.23)	0.480	1.64 (1.49–1.81)	**< 0.001**	1.00 (0.45–2.24)	0.983
pH						
≥ 7.08	Reference		Reference		Reference	
< 7.08^b^	2.34 (1.19–4.58)	**0.014**	1.58 (1.40–1.78)	**< 0.001**	1.09 (0.36–3.26)	0.881
Paco_2_ (mm Hg)						
35–45	Reference		Reference		Reference	
< 35	0.79 (0.33–1.90)	0.593	0.96 (0.81–1.14)	0.636	1.02 (0.41–2.56)	0.962
> 45	0.55 (0.29–1.03)	0.062	1.04 (0.93–1.17)	0.481	0.48 (0.22–1.06)	0.071
Previous ECMO run	1.58 (0.48–5.15)	0.499	2.02 (1.65–2.48)	**< 0.001**	1.92 (0.58–6.42)	0.287

DNC = death by neurologic criteria, ECMO = extracorporeal membrane oxygenation, RRR = relative risk ratio.

a 75th percentile.

b 25th percentile.

Two thousand six hundred fifty-seven patients were excluded from the model due to missing data (primarily pH, Paco_2_, and 5-min Apgar scores); *p* values were derived from Wald tests. Boldface values indicate statistically significant *p* values. Dashes indicate insufficient sample size for statistical analysis.

**TABLE 3.** On-Extracorporeal Membrane Oxygenation Model of Multinomial Logistic Regression Analysis Comparing Death by Neurologic Criteria, Other Non-Death by Neurologic Criteria Deaths, and Transplant With Survivors As the Reference Group

Variables	DNC (*n* = 59)		Other Non-DNC Deaths (*n* = 2724)		Transplant (*n* = 34)	
	RRR	*p*	RRR	*p*	RRR	*p*
Cannulation site						
Cervical	Reference		Reference		Reference	
Central	2.36 (1.31–4.27)	**0.004**	1.75 (1.58–1.93)	< 0.001	2.47 (1.31–4.66)	0.005
Pao_2_ (mm Hg)						
65–162	Reference		Reference		Reference	
< 65^b^	1.21 (0.57–2.59)	0.611	1.03 (0.92–1.15)	0.639	0.36 (0.11–1.22)	0.100
> 162^a^	2.54 (1.42–4.55)	**0.002**	1.66 (1.49–1.85)	< 0.001	1.89 (0.98–3.64)	0.056
(Paco_2_ at 24 hr on ECMO–Paco_2_ pre-ECMO/Paco_2_ pre-ECMO) × 100						
≤ 50%	Reference		Reference		Reference	
> 50%	0.92 (0.39–2.18)	0.845	1.44 (1.28–1.63)	< 0.001	0.55 (0.17–1.80)	0.321
Pump flow (L/min)						
≥ 0.31	Reference		Reference		Reference	
< 0.31^b^	1.70 (0.93–3.13)	0.086	1.06 (0.95–1.18)	0.275	0.29 (0.09–0.94)	0.040
pH						
≥ 7.35	Reference		Reference		Reference	
< 7.35^b^	2.76 (1.52–5.00)	**0.001**	1.79 (1.62–199)	< 0.001	0.64 (0.25–1.66)	0.362
Center volume (*n*/yr)						
< 20	Reference		Reference		Reference	
20–49	0.53 (0.21–1.35)	0.181	0.86 (0.76–0.98)	0.022	0.81 (0.31–2.08)	0.659

DNC = death by neurologic criteria, ECMO = extracorporeal membrane oxygenation, RRR = relative risk ratio.

a 75th percentile.

b 25th percentile.

Three thousand seven hundred seventy-one patients were excluded from the model due to missing data. Boldface values indicate statistically significant *p* values.

## DISCUSSION

In this ELSO registry study of neonates who were treated with ECMO, we examined a contemporary cohort (2010–2023) and found that overall survival was 76%. DNC was rare, occurring in 0.5% of all neonates supported with ECMO, and accounted for 2% of all mortality. Pre-ECMO factors associated with greater RRR of subsequent DNC included pre-ECMO cardiac arrest, cardiac support type, and pH below 25^th^ percentile (7.08). On-ECMO, lower pH after 24 hours, Pao_2_ greater than 75^th^ percentile (162 mm Hg), and central cannulation were also associated with greater RRR of subsequent DNC.

A historical study using neonatal data in the ELSO registry, 2005–2010, indicate that severe cardiopulmonary dysfunction before initiation, including conditions such as significant metabolic acidosis and cardiac arrest before cannulation, was associated with greater odds of neurologic injury (17). Our 2010–2023 ELSO registry dataset suggest that neonates with more severe early physiologic derangement—reflected by very low arterial pH or cardiac arrest before cannulation—may be more likely to receive a subsequent diagnosis of DNC. Whether earlier recognition of deterioration or earlier ECMO initiation could influence this risk remains unknown. Further prospective studies are needed to fully understand the impact of early recognition and prompt deployment of ECMO in mitigating these risks. Similarly, a lower on-ECMO pH was independently associated with DNC, which may indicate persistent systemic compromise; however, causality cannot be inferred from these data.

Most neonatal venoarterial ECMO procedures use the right cervical vessels for peripheral cannulation, with central cannulation via a sternotomy being less common (18–20). An older ELSO registry study using a 2007–2008 cohort identified a higher risk of neurologic injury in children cannulated through the carotid artery compared with central cannulation, regardless of age (21). However, this association was not identified in another ELSO registry study in children supported over the wider period, 1989–2013 (22). In our analysis of the ELSO registry dataset (2010–2023), confined to neonates, we identified higher RRR of DNC in those who underwent central cannulation compared with those cannulated through the neck vessels. Since central cannulation is often used when cardiopulmonary bypass weaning fails in neonates with CHD, this association may be confounded by severity of illness rather than a direct consequence of central cannulation on cerebral blood flow. Furthermore, the association between central cannulation and DNC may reflect underlying illness severity rather than an effect of cannulation approach itself.

In newborns, exposure to high Fio_2_ is associated with poorer outcomes in neonatal resuscitation (23). Also, both hypoxia and moderate hyperoxia studied using the 2015–2020 ELSO registry data are associated with greater odds of mortality in neonatal ECMO (4). In our study, a Pao_2_ greater than 162 mm Hg was independently associated with a higher RRR of DNC, and emerged as an important variable during ECMO associated with DNC. Proposed mechanisms underlying the harm from hyperoxia include the increased formation of reactive oxygen species, leading to oxidative stress and cell death (24). Hyperoxia during ECMO may also indicate a sicker patient population, as poor hemodynamics or impaired perfusion may lead clinicians to use of higher Pao_2_ to augment oxygen delivery (25). However, the observed association between hyperoxia and the RRR of DNC appeared to be independent of ECMO circuit flow, suggesting that the effect may be physiologic and not directly related to poor perfusion. Hyperoxia could, therefore, be regarded as a modifiable and potentially iatrogenic exposure, like in other areas of pediatric critical care (26, 27). Although these considerations need validation through adequately powered ECMO randomized trials, it appears sensible to adjust Fio_2_ on both ventilators and ECMO circuits to maintain Pao_2_ within physiologic ranges in patients appropriately supported by ECMO.

Not surprisingly, our ELSO registry study using neonatal data from 2010 to 2023, reiterates findings in another ELSO neonatal report covering the period 2009–2022 (1): DNC is relatively uncommon in the neonatal ECMO population. However, the prevalence of DNC in our ELSO dataset, of 0.5%, may underestimate the real occurrence of DNC in this population. For example, a review of suspected DNC cases in the United Kingdom, 2015–2023, highlighted the diagnostic challenges in the PICU and neonatal ICU (NICU) populations, noting variability in the extent of diagnostic testing performed, particularly in younger children and NICU patients (28). While the report does not specify whether these patients were supported on ECMO, it underscores the challenging nature of DNC diagnosis in these vulnerable populations. Factors such as sedation, hypothermia, and severe metabolic disturbances can interfere with standard clinical criteria for DNC, such as apnea testing, presenting complex diagnostic scenarios (11). In neonatal ECMO, formal determination of DNC is not always pursued when severe neurologic injury leads clinicians to withdraw support for reasons of poor prognosis rather than to complete DNC testing. In such cases, deaths are typically classified as “other causes,” which may lead to an underestimation of the true occurrence of DNC in registry data (11, 29–32). Improving access to specialized expertise in DNC assessment in very young children may help ensure clearer and more consistent ascertainment and, in some settings, could also support neonatal organ donation pathways (33–35).

Several limitations should be considered when interpreting our analyses. First, we used the ELSO registry data, which is based on voluntary reporting and therefore subject to reporting bias. A key limitation is the ELSO registry’s inherent data granularity, which precluded an in-depth analysis of detailed cardiac arrest events or pre-ECMO neurologic status, thereby limiting the direct clinical applicability for predictive modeling. There is also the problem of repeated and overlapping analyses using the ELSO dataset, without controlling for prior known associations with outcome (14). Second, we did not have access to important data such as the anticoagulation regimen on ECMO, which may have had an important influence on the subsequent progression to DNC. Third, the ELSO registry does not provide information on ABI severity and how and when ABI was diagnosed. In particular, the use of ancillary studies and the compliance with currently accepted U.S. National and American Academy of Pediatrics guidance on determining DNC are not reported. Fourth, the absence of detailed clinical data regarding the direct cause of DNC—such as diffuse cerebral edema or large ICH—limits our ability to characterize the final pathway leading to DNC. Fifth, limitation or withdrawal of circulatory support in children with catastrophic brain injury before such a diagnosis and the fact that some centers may choose to only determine DNC in cases of organ donation may all have led to an underestimation of the primary outcome in our study. Finally, given the limited number of outcome events, including 14 variables across two models may have increased the risk of overfitting and biased estimates, especially for low-prevalence predictors, and residual center-level confounding cannot be entirely excluded despite volume adjustment.

In conclusion, our analysis of the ELSO 2010–2023 neonatal ECMO dataset identified several pre- and on-ECMO factors associated with DNC. These included pre-ECMO cardiac arrest, metabolic acidosis, as well as hyperoxia and lower pH during ECMO. Hyperoxia is a modifiable risk factor that requires careful management. Nonetheless, its relationship with neurologic outcomes is complex, and further investigation is needed to fully understand its role in the development of DNC in neonatal ECMO.

## ACKNOWLEDGMENTS

We thank the Extracorporeal Life Support Organization for technical support. We also thank Antoine Poncet (Clinical Research Center and Division of Clinical Epidemiology, Geneva University Hospitals, Geneva, Switzerland) for help and support with statistical analyses.

## Supplementary Material


