# Supplementary material for: Death by Neurologic Criteria in Neonates Undergoing Extracorporeal Membrane Oxygenation: Extracorporeal Life Support Organization Registry Study, 2010–2023
Source: Pediatr Crit Care Med. 2026 Jan 12;27(3):297–306. doi: 10.1097/PCC.0000000000003891 (PMC12931849; doi:10.1097/PCC.0000000000003891)
Supplement: Supplementary file 1 [file pcc-27-297-s001.pdf]

Supplementary tables

Table of contents

Table S1. ICD-9 and ICD-10-CM Codes for Diagnostic Groups page 2

Table S2. Number of reporting centers per year during the study period page 3

Table S3. Characteristics of blood gas before and during ECMO in the whole study population page 4

Table S1. ICD-9 and ICD-10-CM Codes for Diagnostic Groups

Diagnostic Group	ICD-10-CM Codes	ICD-9-CM Codes
Cardiomyopathies, Myocarditis	I51.4, I40, I41, I42, I43, I25	425, 422.91
Congenital Heart Disease (CHD)	I00-I09, I33-I39, Q20-Q28, I00-I99	745.0-747.29
Meconium Aspiration Syndrome (MAS)	P24.0	770.1
Congenital Diaphragmatic Hernia (CDH)	Q79.0, Q79.1	756.6
Asphyxia of the Newborn	P21.0, P21.9, P84	768.5, 786.6
Persistent Pulmonary Hypertension of the Newborn (PPHN)	P29.3	747.83

Table S2. Number of reporting centers per year during the study period

Year	Number of reporting centers
2010	135
2011	136
2012	151
2013	149
2014	161
2015	169
2016	167
2017	177
2018	193
2019	187
2020	191
2021	178
2022	180
2023	163

Overall, 279 unique centers reported at least one case during the 2010-2023 study period.

Table S3. Characteristics of blood gas before and during ECMO in the whole study population

Variables	Overall (n=14970)	Survived (n=11367)	DNC (n=70)	Other deaths (n=3482)	Transplant (n=51)
**Pre-ECMO Support**					
HFOV	5954 (40)	4787 (42)	20 (29)	1147 (33)	-
Vasoactive support	12994 (87)	9932 (87)	52 (74)	2971 (85)	39 (76)
Surfactant	2729 (18)	2366 (21)	6 (9)	356 (10)	1 (2)
iNO	9445 (63)	7518 (66)	32 (46)	1887 (54)	8 (16)
Neuromuscular blockade	8381 (56)	6425 (57)	32 (46)	1893 (54)	31 (61)
Bicarbonate (mmol/L)	3613 (24)	2586 (23)	21 (30)	994 (29)	12 (24)
Intubation to ECMO time (hours)	60±94	57±89	41±69	69±109	70±120
**Pre-ECMO blood gas**					
pH	7.20 [7.08-7.3]	7.20 [7.09 - 7.31]	7.15 [7.00-7.31]	7.17 [7.02-7.29]	7.30 [7.18-7.39]
PaO2 (mmHg)	38 [28-53]	38 [29–52]	41 [31-76]	39 [28-57]	52 [40-146]
PaCO2 (mmHg)	55 [43-72]	55 [43-72]	50 [39-63]	56 [44-75]	40 [36-47]
Bicarbonates (mmol/L)	21.5 [18.0-25.0]	21.9 [18.2-25.0]	17.1 [14.5-20.8]	21 [16.9-24.3]	20.0 [17.3-23.0]
Lactate (mmol/L)	4.3 [2.2-8.5]	3.9 [2.1-7.6]	11.0 [5.1-14.5]	5.9 [2.8-11.0]	5.2 [3.4-11.3]
SaO2 (%)	71 [49-87]	71 [50-86]	77 [47-92]	69 [44-87]	83 [70-97]
**ECMO flow (L/min)**					
H4	0.36 [0.30-0.44]	0.37 [0.30-0.44]	0.38 [0.29-0.46]	0.36 [0.30-0.45]	0.46 [0.37-0.57]
H24	0.37 [0.31-0.45]	0.37 [0.31-0.45]	0.40 [0.30-0.47]	0.38 [0.31-0.46]	0.46 [0.38-0.58]
**On-ECMO blood gas**					
pH	7.40 [7.35-7.44]	7.40 [7.35-7.44]	7.36 [7.28-7.41]	7.38 [7.32-7.43]	7.42 [7.38-7.46]
PaO2 (mmHg)	98 [65-162]	93 [64-153]	112 [65-194]	115 [68-188]	142 [95-182]
PaCO2 (mmHg)	42 [38-47]	43 [38-47]	41 [37-45]	42 [37-47]	45 [40-47]
Bicarbonates (mmol/L)	26.0 [23.0-29.0]	26.0 [23.2-29.0]	22.0 [19.0-25.4]	25.0 [22.0-28.0]	27.0 [24.5-31.6]
Lactate (mmol/L)	2.1 [1.4-3.4]	2.0 [1.4-3.0]	5.3 [3.0-10.2]	2.7 [1.7-5.3]	2.2 [1.6-3.0]
SaO2 (%)	98 [94-99]	98 [94-99]	98 [94-100]	98 [95-99]	99 [97-100]

Legends: DNC; death by neurological criteria. HFOV; high-frequency oscillatory ventilation. iNO; inhaled nitric oxide. Values are numbers with (%) or medians with [IQR].
